# Linking biodiversity and ecological function through extensive microeukaryotic movement across different habitats in six urban parks

**DOI:** 10.1002/imt2.103

**Published:** 2023-04-17

**Authors:** Shuzhen Li, Kexin Ren, Xue Yan, Andrey N. Tsyganov, Yuri Mazei, Alexey Smirnov, Natalia Mazei, Yiyue Zhang, Christopher Rensing, Jun Yang

**Affiliations:** ^1^ Aquatic EcoHealth Group, Key Laboratory of Urban Environment and Health, Fujian Key Laboratory of Watershed Ecology Institute of Urban Environment, Chinese Academy of Sciences Xiamen China; ^2^ University of Chinese Academy of Sciences Beijing China; ^3^ Lomonosov Moscow State University Moscow Russia; ^4^ Faculty of Biology Shenzhen MSU‐BIT University Shenzhen China; ^5^ A. N. Severtsov Institute of Ecology and Evolution Russian Academy of Sciences Moscow Russia; ^6^ Department of Invertebrate Zoology, Faculty of Biology St. Petersburg University St. Petersburg Russia; ^7^ Key Laboratory of Urban Environment and Health, Institute of Urban Environment Chinese Academy of Sciences Xiamen China; ^8^ Institute of Environmental Microbiology, College of Resources and the Environment Fujian Agriculture & Forestry University Fuzhou China

## Abstract

Highly diverse but divergent microeukaryotes dwell in all types of habitats in urban park ecosystems. Extensive microbial migration occurs between both terrestrial and aquatic habitats. Microbial movement is beneficial to the maintenance of biodiversity and the exchange of functional guilds.

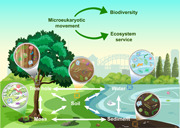

Urban parks, as an important urban greenspace, play essential roles in regulating the local climate and promoting human mental and physical well‐being [1, 2]. Parks are also home to microeukaryotes, which comprise a wide range of morphologically, genetically, and functionally diverse lineages, and are crucial trophic links in microbial food webs and essential for ecosystem function [2]. Previous studies have shown that urban parks are important hotspots for the taxonomic and functional diversity of microeukaryotes [1, 3]. However, most studies performed in urban parks have focused on specific habitats (e.g., soil) and certain microbial groups, including fungi, protists, or testate amoebae [3, 4, 5, 6]. This greatly limits a comprehensive understanding of the biodiversity and distribution of overall microeukaryotes in urban parks. Additionally, soil has usually been the focus of scientific attention, as it harbors the most diverse microbiome on Earth, and soil microbes greatly contribute to the One Health concept, which emphasizes that the health of humans and ecosystems are inseparably linked to the microbiome [7]. In addition to soils, microeukaryotes also dwell in many other adjacent habitats in urban parks, such as mosses, tree holes, water columns, and pond sediments, while these ecological niches remain largely neglected.

Microorganisms can move actively or passively across habitats. The movement of an organism is a fundamental feature of life [8]. The movement allows microorganisms to converge on a favorable environment, encounter resources, and evade predators and plays important roles in determining the diversity and dynamic of microbes at the community level [9]. However, which microbes move, and in particular, what the ecological and evolutionary consequences of cross‐habitat movements are, are poorly understood [8], let alone, refine our understanding of the role of microeukaryotes in human health through cross‐habitat movements. In addition, managing urban microbes requires a predictive understanding of the ecological patterns and processes that mediate how environmental factors shape microbial community assembly and drive migration between different habitats [4]. Until now, the relative role of stochastic and deterministic processes in shaping microeukaryotic communities across urban habitats remains unclear [10]. Given the above background, uncovering the microeukaryotic biodiversity and community composition, the ecological processes that shape metacommunity assembly, and the movement of microeukaryotes between habitats are beneficial for better understanding urban microbial ecology. These insights will further contribute to conserve, restore, and manage urban park ecosystems [2, 8, 11, 12].

High‐throughput sequencing of 18S rRNA genes has been widely implemented to characterize microeukaryotic communities. Among hypervariable regions, both V4 (about 400 bp) and V9 (about 150 bp) regions were most commonly used for biodiversity assessments [13, 14, 15, 16]. Until now, there is no consensus on which region provides better recovery of the microeukaryotes, and contradictory results have been observed in field surveys [13, 16, 17, 18, 19]. Additionally, previous comparisons have mainly focused on specific microeukaryotic groups such as ciliates [17] and cercozoa [20], or limited habitats such as ponds [13] and marine water [19, 21], which hinder a direct comparison across studies and may have affected our understanding of the microeukaryotic diversity and movement profile.

We currently lack a comprehensive perspective on the microeukaryotes of urban parks. Understanding the biodiversity and potential movement of microeukaryotes across different habitats will help to understand their functions in controlling the health of urban environments. To achieve these goals, samples were collected across different habitats including moss, soil, tree hole, water column, and pond sediment. We aimed to address the following questions: (1) What are the similarities and differences in biodiversity and community structure of microeukaryotes between habitats? (2) What are the community assembly driving force and migratory routes of microeukaryotes between habitats, and what are the effects of microbial movement on biodiversity and functions of specific groups? (3) Whether performing V4 and V9 region sequencing data will provide similar diversity patterns? To our knowledge, this is the first analysis focusing on the cross‐habitat movement of microeukaryotes and its effect on biodiversity and ecosystem function in urban park ecosystems.

## RESULTS

### Community composition in urban parks

**Figure 1:**
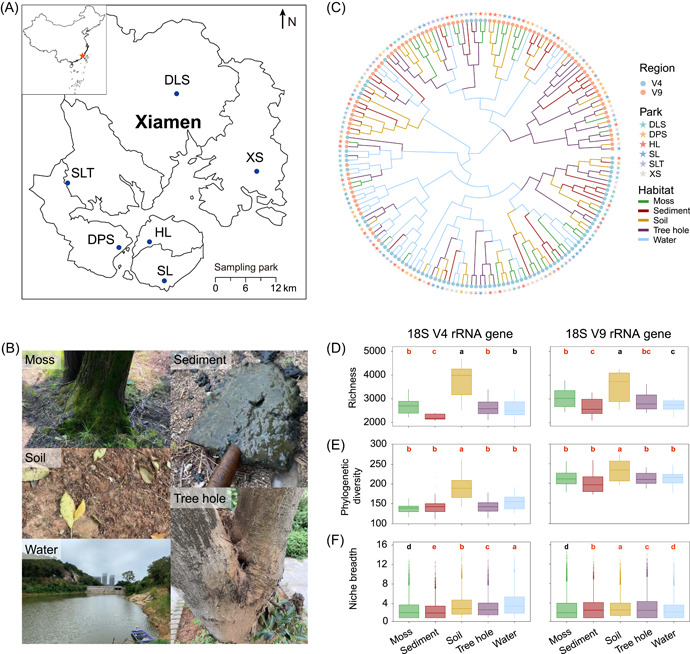
Urban park habitats and their microeukaryotic community α‐diversity. (A) Locations of sampling parks in Xiamen city (DLS, Dalunshan park; DPS, Dapingshan park; HL, Huli park; SL, Shangli park; SLT, Shuanglongtan park; and XS, Xiangshan park). The red star on the map shows the location of Xiamen. (B) Five sampling habitats, including moss, sediment, soil, tree hole, and water. (C) Hierarchical cluster of 180 samples containing six parks, five habitats, and two amplified regions with three replicates. (D–F) α‐Diversity (zOTU richness, phylogenetic diversity, and niche breadth) of microeukaryotic communities in five habitats based on V4 and V9 regions of 18S rRNA genes, respectively. The statistical method used is the Wilcoxon test performed in R and different letters indicate a significant difference (*p* < 0.05) between the two habitats. Red letters indicate a significant difference (*p* < 0.05) between the V4 and V9 regions.

In our study, microeukaryotes from five habitats, that is, moss, soil, tree hole, water, and sediment, were evaluated (Figure 1A,B). Rarefaction curves tended to be flat indicating that the sequencing depth was sufficient (Supporting Information: Figure S1). Permutational multivariate analysis of variance and hierarchical clustering clearly indicated that amplicon regions contributed most of the differences in the recovered community, followed by habitat types, and different parks had the least effect (Figure 1C and Supporting Information: Table S1). The highest taxonomic and phylogenetic diversities based on zero‐radius operational taxonomic units (zOTUs) were observed in soil (Figure 1D,E). Notably, microeukaryotes recovered using the V9 region were more diverse at both taxonomic and phylogenetic levels than those using the V4 region. The niche breadth was the greatest in water samples when estimated by the V4 region and the lowest by the V9 region, thereby indicating a controversial result provided by different primer pairs in aquatic environments (Figure 1F).

For V4 and V9 regions, 2219 and 3987 zOTUs were observed in all habitats, respectively (Supporting Information: Figure S2A,B). The smallest or largest number of unique microeukaryotes was determined to be in water by sequencing the V4 or V9 regions, respectively. More Opisthokonta, Excavata, Stramenopiles, and unassigned supergroups, as well as fewer Alveolata, Amoebozoa, and Rhizaria were shown using the V9 region than the V4 region (Supporting Information: Figure S2C,D). The relative abundances of Stramenopiles were higher in sediments, whereas Hacrobia were more abundant in water samples (Supporting Information: Figure S2E,F). Consistently, Opisthokonta was the most abundant supergroup in all habitats. Within Opisthokonta, the V4 region detected more fungal taxa with different trophic modes than the V9 region, while a higher abundance of pathotrophs was observed using the V4 region, higher abundances of saprotrophs and symbiotrophs were identified using the V9 region (Supporting Information: Figure S3A,C). Soil and sediment had the most and fewest fungal guilds in both number and abundance, respectively (Supporting Information: Figure S3B,D). Specifically, relative abundances of testate amoebae, which belonged to Amoebozoa, were summarized. Seven genera were observed by sequencing the V4 region, and there was a high correlation (*r* > 0.9, *p* < 0.05) of relative abundance between microscopic analysis and high‐throughput sequencing. On the other hand, only *Euglypha* was found in the V9 region‐based community (Supporting Information: Table S2).

### Community dissimilarity and assembly

**Figure 2:**
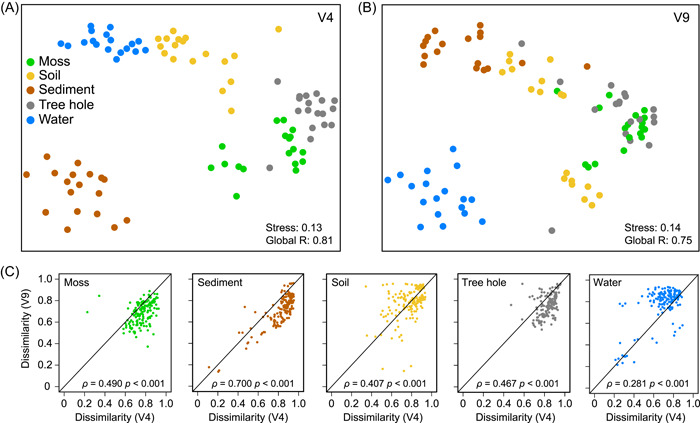
Comparison of microeukaryotic community β‐diversity across five habitats. Nonmetric multidimensional scaling (NMDS) ordination of microeukaryotic communities based on the Bray–Curtis dissimilarity for the V4 (A) and V9 (B) regions. (C) Spearman's correlation of Bray–Curtis dissimilarity between V4 and V9 region‐based microeukaryotic communities. Correlations are calculated as pairwise comparisons of all dissimilarity matrix data, the *ρ* value indicates the correlation coefficient, and the black line is *y* = *x*.

Nonmetric multidimensional scaling (NMDS) analysis clearly demonstrated specific community distinctions between habitats (Figure 2A,B). The spatial patterns of microeukaryotes based on both the V4 and V9 regions exhibited a remarkable similarity in moss, soil, pond sediment, and tree hole (*ρ* = 0.407–0.700, *p* < 0.001), whereas it was low in the water sample (*ρ* = 0.281, *p* < 0.001) (Figure 2C). The community variation within each habitat type also greatly varied (*p* < 0.05) between habitats or sequencing regions, and the lowest and highest dissimilarity was observed from water in the V4 and V9 regions, respectively (Supporting Information: Figure S4A,B). Turnover was much more important than nestedness in shaping communities (Supporting Information: Figure S4C,D).

For a single habitat, the fit for the neutral community model (*R* ^*2*^) was moss > water > tree hole > pond sediment > soil, and there was a significant decrease in model *R* ^*2*^ value with increasing habitat number (Supporting Information: Figure S5). Furthermore, dispersal limitation was the main driving factor in the community assembly process in a single or multiple habitats (Supporting Information: Figure S6).

### Microeukaryotic movement across habitats

**Figure 3:**
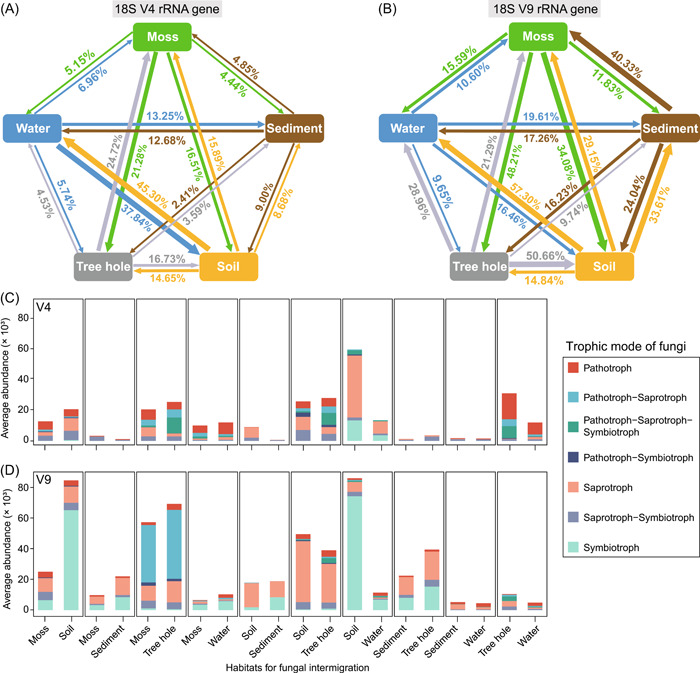
Connectivity of microeukaryotes across different habitats and potential migrated fungal taxa. Fast expectation‐maximization microbial source tracking analysis for V4 (A) and V9 (B) regions at the zero‐radius operational taxonomic unit (zOTU) level. The direction of the arrows represents the source‐sink relationships, and percentages represent the contribution that each source provides. Total relative abundance of potential migrated fungal taxa with the assigned trophic mode in each pair of habitats from V4 (C) and V9 (D) regions. Wilcoxon test is performed in R to verify whether a zOTU's abundance significantly differs between each pair of habitats. We assume that species may have migrated between the two habitats if there is no significant difference in the abundance of the same fungal species in these two habitats. The *x*‐axis is each habitat pair. The trophic mode is identified using FUNGuild.

Consistently, soil had the highest contribution to water for the V4 (45.30%) and V9 (57.30%) region‐based results (Figure 3A,B) and was identified as the highest proportion of source microorganisms to its surroundings (Supporting Information: Figure S7A). In the V4 region‐based communities (Figure 3A), about 37.84% of the soil microeukaryotes were likely inherited from the water, and 45.30% of water microeukaryotes came from soil that indicate high migration rates between aquatic and terrestrial habitats. Moderate connectivity (around 12%–24%) was detected between aquatic (sediment and water) habitats or among aboveground terrestrial (moss, soil, and tree hole) habitats. For the V9 region‐based communities (Figure 3B), tree hole and moss contributed greatly to soil microeukaryotes (50.66% and 34.08%, respectively). The soil had a high representation of the microeukaryotic group contributed to sediment (33.61%) and water (57.30%), whereas sediment served as a source for soil (24.04%) and moss (40.33%) microeukaryotes. Overall, communities detected using the V9 region identified a significantly (*p* < 0.05) higher percentage of clear sources than those using the V4 region in most habitats (Supporting Information: Figure S7B).

To gain a deeper understanding in microeukaryote movement (or connectivity) in urban parks, we first identified shared taxa in each pair of habitats, and significant (*p* < 0.05) differences in niche breadth on these shared taxa were observed between habitat pairs (Supporting Information: Figure S8). This indicated that the niche breadth of the same species across habitats differed, that is, the number of individuals should be different. Based on this, we inferred the taxa may migrate if the abundance of the same species displayed no significant difference between habitats. Opisthokonta was most likely to travel between habitats and accounted for half of all migrated taxa, followed by Alveolata, Archaeplastida, and Rhizaria (Supporting Information: Figure S9A−D). Interestingly, we found that specific taxa from one habitat migrated to different adjacent habitats (Supporting Information: Figure S9E,F). For instance, Archaeplastida were the main taxa transferred between soil and sediment, and Rhizaria were more prone to migrate from soil to moss and tree holes. Overall, V9 region‐based microeukaryotes had more migrated taxa numbers and relative abundances than the V4 region. Considering the diverse trophic modes of fungi, dozens of fungal taxa were estimated to have moved between habitats, including pathotrophs, saprotrophs, and symbiotrophs (Supporting Information: Figure S10). Notably, pathotrophs were more prone to migrate between moss and tree holes, as well as tree holes and water (Figure 3C,D). A large number of saprotrophs and symbiotrophs moving from soil to water were detected by V4 and V9 regions, respectively.

## DISCUSSION

### Microeukaryotic movement contributes to the maintenance of biodiversity

Terrestrial habitats, including moss, soil, and tree hole, harbored higher α‐diversity than aquatic habitats (i.e., water and sediment) in urban parks (Figure 1) in line with a previous study focusing on bacterial and protistan communities [22]. Differentiated microeukaryotes dwell in adjacent habitats with a high degree of dissimilarity (β‐diversity) (Figure 2 and Supporting Information: Figure S4), indicating each habitat contributes to the total diversity (γ‐diversity) of the urban park ecosystems.

Complex source‐sink relationships were observed between these adjacent environments (Figure 3), hinting at extensive microeukaryotic travel routes in urban parks. Soil is the largest reservoir of microbial diversity on Earth, and unsurprisingly, we found that soil contained the highest diversity and contributed the highest microeukaryotic source to surrounding habitats, especially for the aquatic ecosystem. At the same time, various habitats including moss, tree hole, sediment, and water, which have long been ignored, also harbor comparable biodiversity and extensive microbial exchange, as well as cover most of the open spaces available for human recreational activities. This suggests that any habitat has the potential to be a microeukaryotic source. This is beneficial for biodiversity maintenance as sinks can become the source and vice versa when environmental conditions fluctuate to maintain a community of eukaryotes [23].

Urbanization has been shown to cause homogenization of the soil microbiome and reduce soil microbial diversity [1]. From our results, the turnover component, instead of nestedness dominated the total community dissimilarity (Supporting Information: Figure S4C,D), further suggesting that the protection of biodiversity in all habitats is equally important [24]. Therefore, we suggest more attention should be paid to the diverse habitats in urban parks and investigate the community exchange profile, to better serve the One Health idea.

### Microeukaryotic movement facilitates the spread of microbes and functions

Spatial patterns of microeukaryotic communities were jointly shaped by niche selection (habitat) and geographical distance (park) (Figures 1C and 2). Both deterministic and stochastic processes were important in shaping the microeukaryotic community within a habitat, and deterministic dispersal limitation across different kinds of habitats leads to community turnover (Supporting Information: Figures S5 and S6).

Our analysis uncovered potential migrations between urban park habitats. Many connections are explicable when considering environmental mass flows as a factor contributing to the spread of microorganisms. For example, soil as a source of aquatic life is a logical assumption, given the runoff of rainwater. The same applies to the exchange of tree holes and moss with soil. Although water and sediment are closely tied in aquatic ecosystems, the traceable proportions between them were lower than 20% (Figure 3), confirming the conclusion that microeukaryotes from water and sediment were distinct [25]. Still, over half of the community members could not be identified with definite sources, this may be partly due to the existence of unique species in each habitat, or a significant part of species remains recovered only at the molecular level. Species may actively move or be passively carried and distributed by human activities, wildlife, winds, rains, or other unknown disturbances [26]. Understanding the driving forces of microbial movement is very important, as it can help us understand how species are distributed in ecosystems seeded with microbes and clarify the role of environmental selection in urban ecosystems from the fundamental statement that “everything is everywhere” [27].

We further estimated the potentially migrating taxa across habitats (Figure 3). Opisthokonta was the main group moving between habitats, and pathotrophs were frequently estimated to be traveling between moss, tree hole, and water, indicating that these habitats may pose higher risks to human health. Saprotroph and symbiotroph guilds undertake important functions such as organic decomposition and biochemical cycling, which play important roles in ecosystem functions [28]. They are estimated to move in all kinds of habitats generally, and soil and water had the most active communication. Admittedly, this analysis, based on the similarity of taxa abundances, has some limitations. On one hand, species may have similar individual numbers between habitats but might not have derived from migration. For example, long distances and huge obstacles between habitats could make it impossible for species migration; additionally, some biological factors (e.g., interaction and priority effect) might change the individual numbers [29]. On the other hand, species can maintain large or small populations in two habitats after migration when facing a competitive advantage or disadvantage, respectively. This is a very important mechanism for species coexistence in metacommunities, which is called the source‐sink effect [23, 30]. Overall, despite the inevitable false positives and false negatives, we attempted to investigate the microbial source from proportions to migration type and abundance. In addition, we found a consistent pattern, that is, identified that the number and abundance of migrated taxa were low when the source‐sink transfer percentage was low (Figure 3A,B and Supporting Information: Figure S9). Further research is expected to develop new methods through movement ecology for a better understanding of the causes, mechanisms, and consequences of migrated communities and for generating practical strategies for managing ecosystem benefits in cities.

### Comparison of microeukaryotic communities between the V4 and V9 regions

We identified a higher taxonomic and phylogenetic diversity based on the V9 region (Figure 1), and this is consistent with previous studies [13, 18, 19]. However, many microeukaryotes identified by the V9 region belonged to unassigned taxa (Supporting Information: Figure S2), indicating there may be biases in the V9 primer coverage or database. By identifying the taxa number and correlation between microscopic analysis and high‐throughput sequencing of testate amoebae, the results clearly showed that the V4 region was more accurate than the V9 region in this group (Supporting Information: Table S2).

Notably, the primer selection had a higher influence on the microeukaryote in aquatic habitats (water and sediment) compared with terrestrial habitats (moss, soil, and tree hole) (Figures 1 and 2). Many studies have utilized different hypervariable regions to assess microeukaryotic diversity, for instance, two large circumglobally oceanographic expeditions, Malaspina 2010 [31] and TARA Oceans [14] amplified the V4 and V9 regions, respectively. Our analyses indicate that care should be taken in primer selection when investigations are focused on microeukaryotes from aquatic ecosystems and further comparisons with related terrestrial ecosystems, as the diversity may be selectively and differently recovered by different primer pairs.

## CONCLUSION

Our work highlights that highly diverse but divergent microeukaryotes dwell in all types of habitats, and extensive microbial migration can occur between both terrestrial and aquatic habitats. Microbial movement is beneficial to the maintenance of biodiversity and the exchange of functional guilds, while the latter may have beneficial or adverse consequences for human beings and the environment. Future studies on the urban microbiome are encouraged to pay more attention to multihabitat and practice the One Health concept in the three‐dimensional space of urban greenspaces, to better connect human health to the health of microbes and the environment.

## METHODS

### Sampling

Samples were collected in six urban parks in July 2020 in Xiamen city (24.48 N, 118.08 E). In each urban park, five adjacent habitats (moss, soil, tree hole, pond surface water, and pond surface sediment) were sampled in three replicates, thereby resulting in a total of 90 samples. Detailed descriptions of sample processing, sequencing, and sequence quality control are summarized in the Supporting Information.

### Data analysis

Sequencing reads were assigned to zOTUs generated by the UNOISE3 algorithm [32]. There were 15,112 zOTUs with 86,235 sequences per sample for the V4 region and 36,726 zOTUs with 206,353 sequences per sample for the V9 region. zOTUs having fewer than 10 sequences were discarded to ensure rare species were not the result of sequencing errors [33]. To facilitate the comparison of the results obtained by different primer sets based on the same sequences per sample [34, 35], we normalized the sequencing effort to 86,235 sequences from each sample for both V4 and V9 region‐based communities. Finally, there were 15,112 and 15,440 zOTUs for the V4 and V9 regions, respectively.

The taxonomic affiliations were based on the Protist Ribosomal Reference database (PR^2^) (Release 115) [36]. Fungi were retrieved and fungal guilds were identified using FUNGuild v1.2 [28]. Specifically, testate amoebae, a kind of widely distributed protists living in soil and freshwater, were summarized at the genus level, to explore the accuracy of different primers compared with microscopic analysis based on the same samples [6]. Other statistical analyses are described in Supporting Information.

### Connectivity of migrating microeukaryotes among habitats

Different environments can select different species. We further assume that the niche breadths of the same species in different environments are different, which leads to different individual numbers. If this assumption is verified, we believe that individuals of a species may have migrated between the two habitats if there is no significant difference in the abundance of the species between these two habitats. Therefore, we first screened the zOTUs that were present in more than half of the samples in 10 pairs of habitats and then assumed that these zOTUs can theoretically exist in this pair of habitats. Subsequently, the Wilcoxon paired test was used to test whether there were significant differences in niche breadth from shared species in each habitat pair. Then, the migrated species were estimated based on the similar zOTU abundance between this pair of habitats.

## AUTHOR CONTRIBUTIONS

**Shuzhen Li**: Conceptualization; methodology; software; formal analysis; writing—original draft; writing—review and editing; visualization. **Kexin Ren**: Conceptualization; methodology; investigation; writing—review and editing. **Xue Yan**: Validation; investigation; writing—review and editing; visualization. **Andrey N. Tsyganov**: Writing—review and editing. **Yuri Mazei**: Writing—review and editing. **Alexey Smirnov**: Writing—review and editing. **Natalia Mazei**: Writing—review and editing. **Yiyue Zhang**: Writing—review and editing. **Christopher Rensing**: Writing—review and editing. **Jun Yang**: Conceptualization; resources; writing—review and editing; supervision; project administration; funding acquisition.

## CONFLICT OF INTEREST STATEMENT

The authors declare no conflict of interest.

## Supporting information

Supporting information.

## Data Availability

All raw sequences from this study have been deposited in the public NCBI Sequence Read Archive (SRA) with accession numbers PRJNA757135 (V4 region, SRR15652301‐SRR15652390, https://www.ncbi.nlm.nih.gov/bioproject/?term=PRJNA757135) and PRJNA753659 (V9 region, SRR15435537‐SRR15435626, https://www.ncbi.nlm.nih.gov/bioproject/?term=PRJNA753659), as well as NODE database (https://www.biosino.org/node/) (V4 region, OEP002253; V9 region, OEP001878). Some of the vector graphics come from Vecteezy.com. The code scripts used for analysis and visualization have been deposited on GitHub (https://github.com/Listen-Lii/MovementOfMicroeukaryotes). Supporting Information (figures, tables, scripts, graphical abstract, slides, videos, Chinese translated version, and updated materials) may be found in the online DOI or iMeta Science http://www.imeta.science/.
