# Supplementary material for: Linking biodiversity and ecological function through extensive microeukaryotic movement across different habitats in six urban parks
Source: Imeta. 2023 Apr 17;2(2):e103. doi: 10.1002/imt2.103 (PMC10989963; doi:10.1002/imt2.103)
Supplement: Supplementary file 1 — Supporting information. [file IMT2-2-e103-s001.pdf]

Supplementary information

Linking biodiversity and ecological function through extensive microeukaryotic movement across different habitats in six urban parks

Running title

Movement of microeukaryotes between habitats in parks

Shuzhen Li[1,#], Kexin Ren[1,#], Xue Yan[1,2], Andrey N. Tsyganov[3], Yuri Mazei[3,4,5], Alexey Smirnov[6], Natalia Mazei[3], Yiyue Zhang[7], Christopher Rensing[1,8], Jun Yang[1,*]

[1] Aquatic EcoHealth Group, Key Laboratory of Urban Environment and Health, Fujian Key Laboratory of Watershed Ecology, Institute of Urban Environment, Chinese Academy of Sciences, Xiamen, China

[2] University of Chinese Academy of Sciences, Beijing, China

[3] Lomonosov Moscow State University, Moscow, Russia

[4] Faculty of Biology, Shenzhen MSU-BIT University, Shenzhen, China

[5] A.N. Severtsov Institute of Ecology and Evolution, Russian Academy of Sciences, Leninskiy Ave. 33, Moscow, Russia

[6] Department of Invertebrate Zoology, Faculty of Biology, St. Petersburg University, Universitetskaya embankment, 7/9, St Petersburg, Russia

[7] Key Laboratory of Urban Environment and Health, Institute of Urban Environment, Chinese Academy of Sciences, Xiamen, China

[8] Institute of Environmental Microbiology, College of Resources and the Environment, Fujian Agriculture & Forestry University, Fuzhou, China

[#] These authors contributed equally to this study.

Correspondence

Jun Yang, Aquatic EcoHealth Group, Key Laboratory of Urban Environment and Health, Fujian Key Laboratory of Watershed Ecology, Institute of Urban Environment, Chinese Academy of Sciences, Xiamen 361021, China

E-mail: jyang@iue.ac.cn

This supplementary information contains:

- 19 pages
- 1 Supporting Methods
    - Sample processing
    - DNA extraction and sequencing
    - Sequence quality control
    - Statistical analyses
- 10 Figures
- 2 Tables
- 17 References

Supporting Methods

Sample processing

Six urban parks, including Shuanglongtan park (0.55 square kilometer), Dapingshan park (1.50 square kilometer), Huli park (0.11 square kilometer), Shangli park (0.48 square kilometer), Dalunshan park (0.36 square kilometer), and Xiangshan park (10.38 square kilometer) were sampled in July 2020. The moss, surface soil (top 5 cm), and surface sediment (5 cm) were scooped up by a trowel. The litter or detritus in the tree hole, including dust and the small tree bark, were collected. The sample represented 100 g of substrates or 2.5 L of surface waters (0.5 m), which were preserved in ice boxes before treatment in the laboratory. The water samples (500 mL) were pre-filtered through 200 μm mesh to remove larger particles and metazoans and subsequently filtered through a 0.22 μm pore-size polycarbonate filter (47 mm diameter, Millipore, Billerica, MA, USA). 2 g of moss was suspended in 40 mL phosphate buffer saline (PBS), and shaken for 2 h under 180 rpm at 30 °C. The solution was treated with ultrasound for 10 minutes, then left to stand for 2 h. After that, microbes were filtered through a 0.22 mm pore-size polycarbonate filter. All samples were stored at -80 °C until DNA extraction.

DNA extraction and sequencing

For sediments, soils, and tree holes, 0.5 g samples were used to extract DNA. For moss and water samples, filters were cut into small pieces with a sterilized cutter. The total DNA was extracted using the Fast DNA SPIN Kit and the Fast Prep Instrument (MP Biomedicals, Solon, OH, USA) following the manufacturer's instructions. For the whole microeukaryotic community, the V4 or V9 region of the eukaryotic 18S rRNA gene was amplified using the primer pair 547F/967R [1] or 1380F/1510R [2], respectively. A 30 μL polymerase chain reaction (PCR) reaction included 15 μL of Phusion High-Fidelity PCR Master Mix (New England Biolabs, Beverly, MA, USA), 0.2 μM of each primer, and 10 ng of DNA. For the V4 region, the PCR reaction comprised of an initial denaturing step at 95°C for 5 min, followed by 30 cycles of 94°C for 30 s, 45°C for 45 s, and 72°C for 60 s. For the V9 region, the PCR protocol employed an initial denaturing step at 95°C for 5 min, followed by 30 cycles of 95°C for 30 s, 55°C for 30 s, and 72°C for 30 s. At the end of the amplification, the amplicons were subjected to a final 10-min extension at 72 °C. The PCR reaction was performed in triplicate per sample, and then PCR products from triplicate reactions were pooled and gel-purified. All libraries were sequenced on the Illumina platform (Illumina Inc., San Diego, CA, USA) using a paired-end (2 × 250 bp for V4 region; 2 × 150 bp for V9 region) strategy.

Sequence quality control

Paired-end reads were assigned to each sample based on their unique barcodes. Quality filtering was performed by eliminating the low-quality reads with QIIME V1.9.1 [3]. Default settings for quality control processing were used: the maximum number of consecutive low-quality base calls allowed before truncating a read = 3, the minimum number of consecutive high-quality base calls to retain read = 0.75; last quality score considered low quality = 3; the maximum number of ambiguous characters allowed in a sequence = 0. The chimera sequences were detected and removed by using the UCHIME *de novo* algorithm [4].

Statistical analyses

All samples from V4 and V9 regions were merged at the supergroup level, and hierarchical clustering using the "ward.D2" method based on Bray-Curtis dissimilarity was implemented using R software (version 4.1.0) [5] and visualized by the "ggtree" package [6]. Permutational multivariate analysis of variance (PERMANOVA) at the supergroup level was conducted to explore the relative contribution of amplicon region, park, and habitat to the difference at the community level. Rarefaction curves were calculated based on individual samples and each of the habitats to explore whether the sequencing depth was sufficient to cover the majority of microeukaryotic taxa. Indices of α-diversity, including richness and phylogenetic diversity (PD) were calculated. Niche breadths of microeukaryotic groups were estimated through the Levins' niche breadth index by the "spaa" package [7]. Ecological niche breadth of a species represents its ability to utilize a range of resources, and a wider niche breadth is generally considered to have higher metabolic flexibility [8]. Non-metric multidimensional scaling (NMDS) ordination and analysis of similarities (ANOSIM) were used to investigate differences in community compositions among different groups (five habitats and six parks).

The community compositions between samples were analyzed based on the Bray-Curtis dissimilarity. These analyses were performed by the "vegan" [9], "picante" [10], and "ape" [11] packages. Spearman correlation of Bray-Curtis dissimilarity was used to test the significance of the correlation between V4 and V9-based community compositions at different habitats. The overall β-diversity between any two habitats was partitioned into turnover and nestedness components by the "betapart" package [12]. The significance test between different habitats or sequencing regions (V4 *vs* V9) was performed by the Wilcoxon test.

We evaluated the fit of the Sloan neutral community model for microeukaryotic community compositions to determine the potential importance of neutral processes on

community assembly [13]. The value *Nm* determines the correlation between occurrence frequency and regional relative abundance, with *N* indicates the community size and *m* being the immigration rate. The R^2 represents the overall fit to the neutral model. Models were run following a published R script [14,15]. To further infer the ecological assembly processes, we calculated the β-nearest taxon index (βNTI) [16]. $\beta NTI > 2$ indicates variable selection of deterministic process; $\beta NTI < -2$ indicates homogeneous selection of the deterministic process. If the $|\beta NTI| < 2$, the stochastic processes are deemed to drive observed differences. Then, community assembly was further distinguished using the modified Raup-Crick index (RC_{Bray}), which is able to disentangle dispersal limitation (indicated by $RC_{Bray} > 0.95$) and homogenizing dispersal (indicated by $RC_{Bray} < -0.95$). If the $|RC_{Bray}| < 0.95$, an “undominated” fraction, which contains weak selection, weak dispersal, diversification, and drift shaped community structure. FEAST analysis uses an expectation-maximization algorithm and can track the contribution of numerous potential source environments simultaneously [17]. Here, the five habitats in pairs from six urban parks were selected as unknown sinks and potential sources, respectively. There were 18 replicates for each habitat (3 replicates for each habitat in one park × 6 parks), hence, there were 18 replicates for each sink-sources matrix. The input file was prepared under the guidance of the FEAST package, and 1000 iterations were used to perform FEAST analysis.

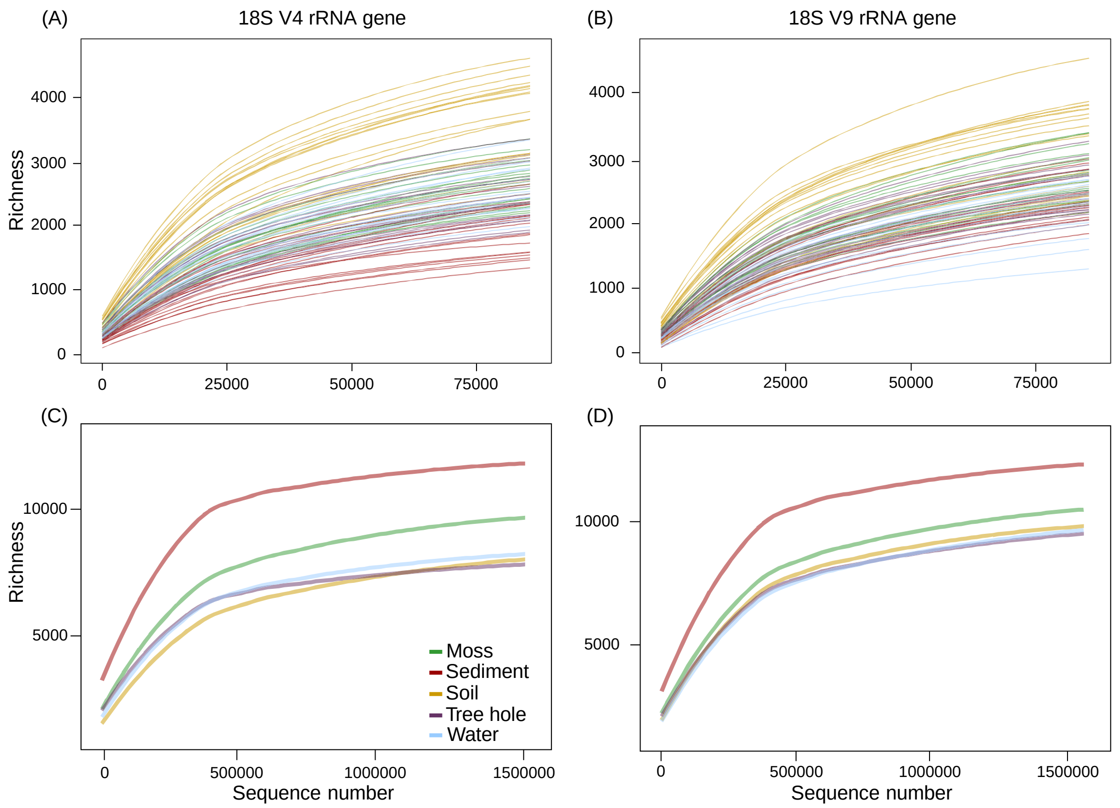

Figure S1 Rarefaction curves of microeukaryotic community based on V4 and V9 regions of 18S rRNA gene, respectively. (A, B) The individual samples; (C, D) The combined sets of five-habitat samples.

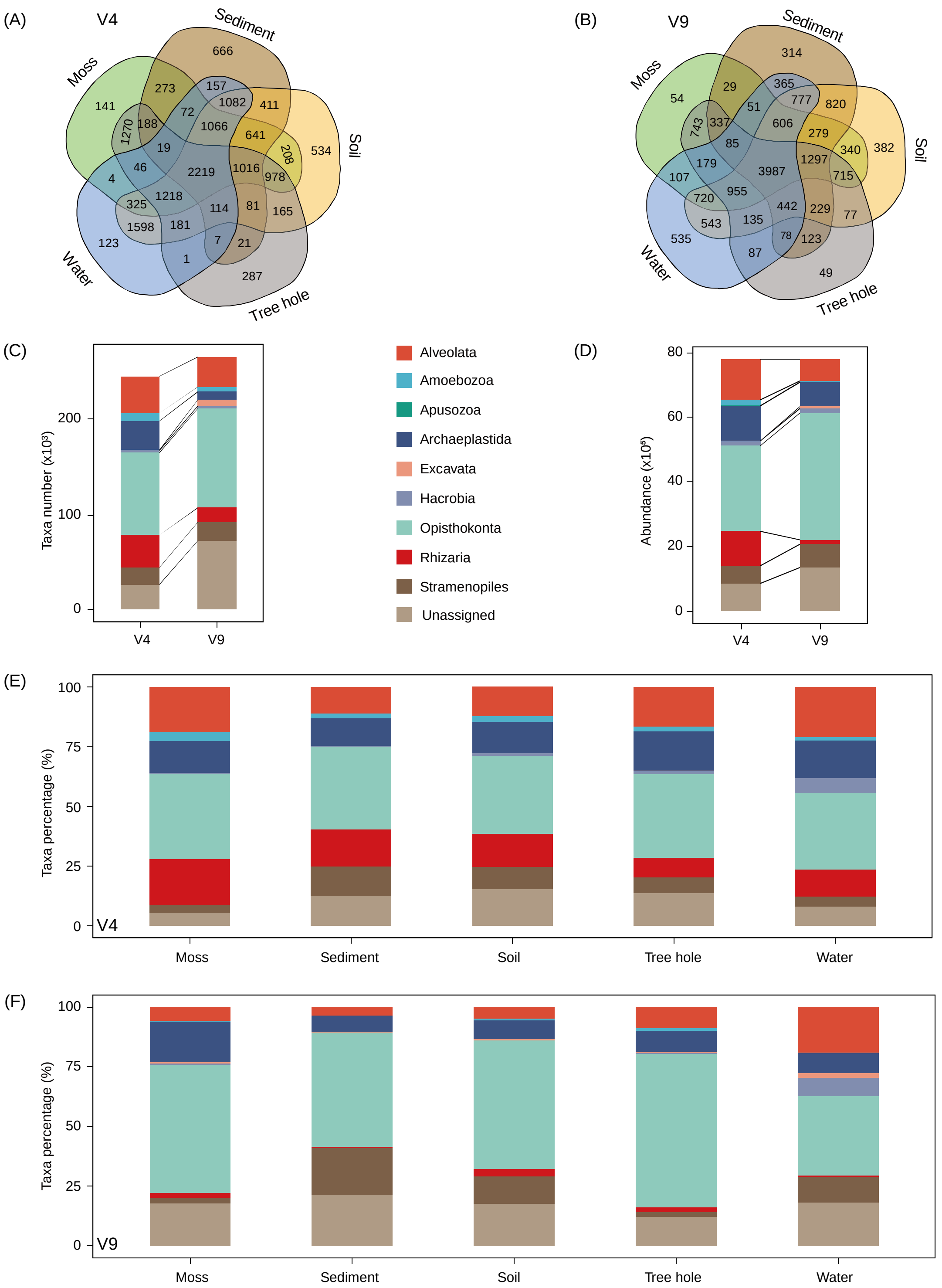

Figure S2 Shared and unique microeukaryotic zOTUs and community composition based on V4 and V9 regions of the 18S rRNA gene. Venn diagrams showing the common and exclusive microeukaryotic taxa for different habitats based on 18S rRNA gene V4 (**A**) and V9 (**B**) regions, respectively. Taxa numbers (**C**) and relative abundances (**D**) of microeukaryotic supergroup. Microbial community composition based on 18S rRNA gene V4 (**E**) and V9 (**F**) regions among five habitats. Values are the relative abundance of microeukaryotic supergroups in each of the five habitats, including moss, sediment, soil, tree hole, and water.

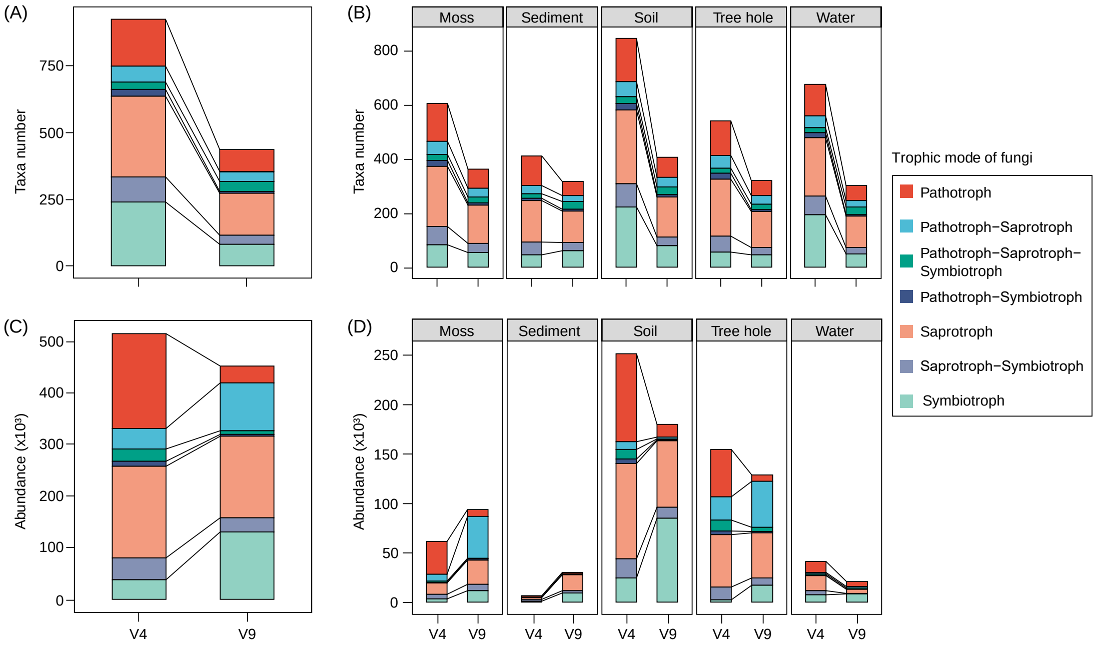

Figure S3 Fungal community composition with different trophic modes based on V4 and V9 regions of 18S rRNA gene. (A) Overall fungal taxa numbers and (B) fungal taxa numbers within each habitat. (C) Overall fungal sequence abundances and (D) fungal sequence abundances within each habitat.

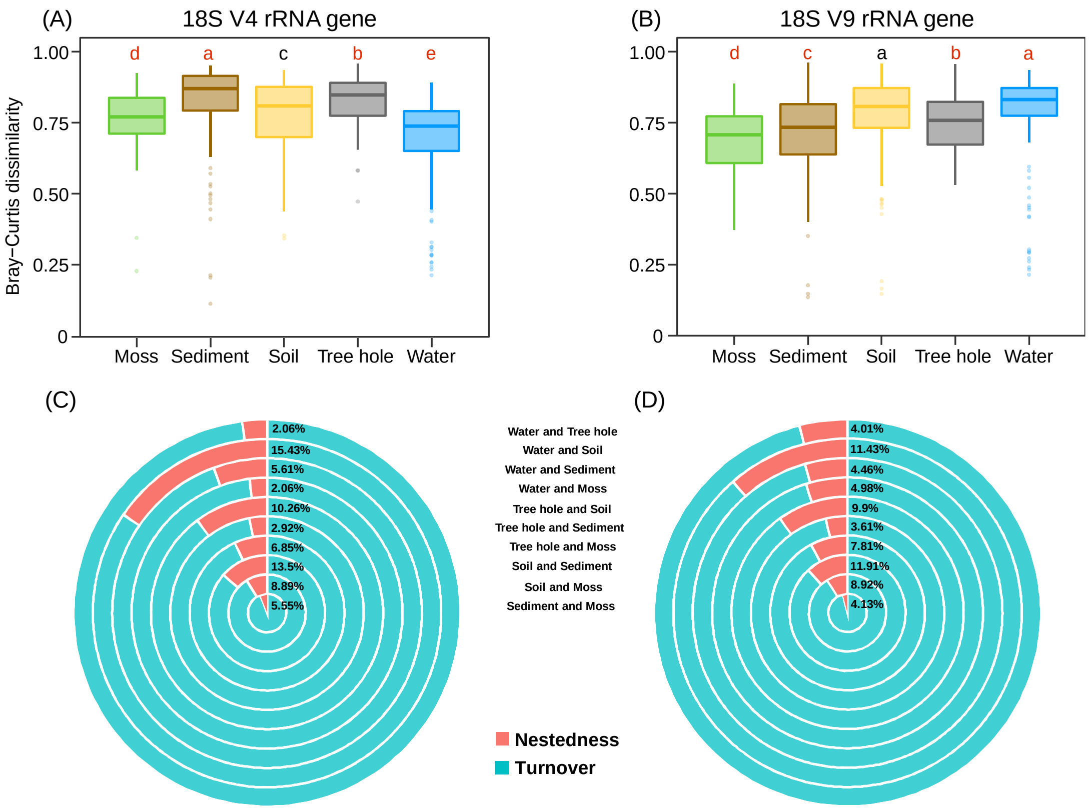

Figure S4 Community dissimilarity within and between habitats. Community dissimilarity from different habitats based on 18S rRNA gene V4 (**A**) and V9 (**B**) regions, respectively. The statistic method used is the Wilcoxon test performed in R and different letters indicate significant difference ($p < 0.05$) between the two habitats. Red letters indicate significant difference ($p < 0.05$) between the V4 and V9 regions. Partitioning β diversity into nestedness and turnover components for V4 (**C**) and V9 (**D**) region-based communities. Values are percentages of nestedness.

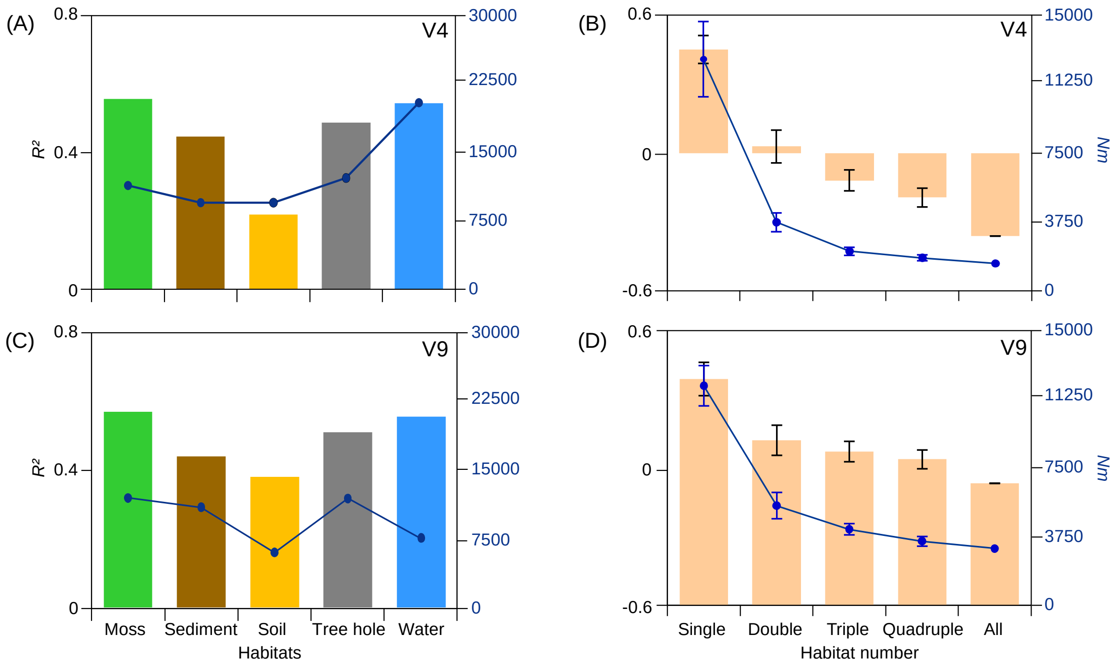

Figure S5 Fit of the neutral community model of microeukaryotic community from urban parks. The predicted occurrence frequencies along the different habitats. Single (**A**) and multi-habitat (**B**) based on V4 region-communities; Single (**C**) and multi-habitat (**D**) based on V9 region-communities. R^2 indicates the fit to the neutral model and is shown in the bar plot. Negative R^2 values can occur when there is no fit to the model. *Nm* indicates the community size time immigration and is shown in a line chart.

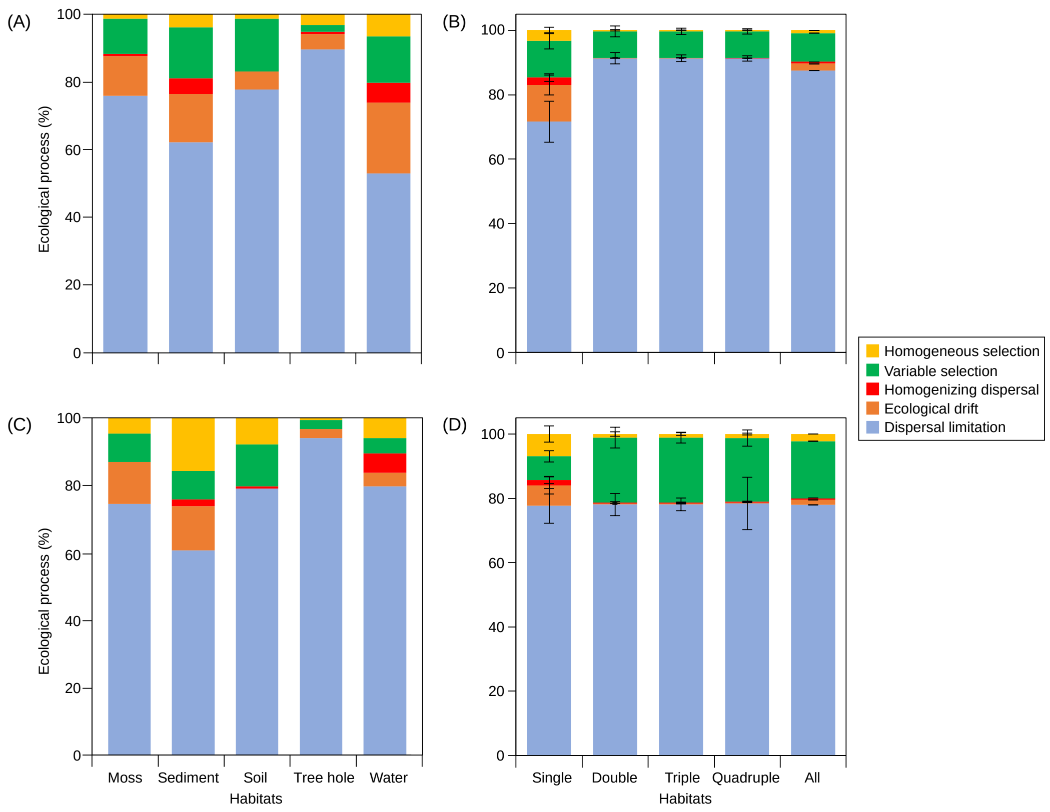

170

171 **Figure S6 Ecological processes shaping the microeukaryotic community.** Single (**A**)
172 and multi-habitat (**B**) based on the V4 region; Single (**C**) and multi-habitat (**D**) based
173 on the V9 region, respectively.

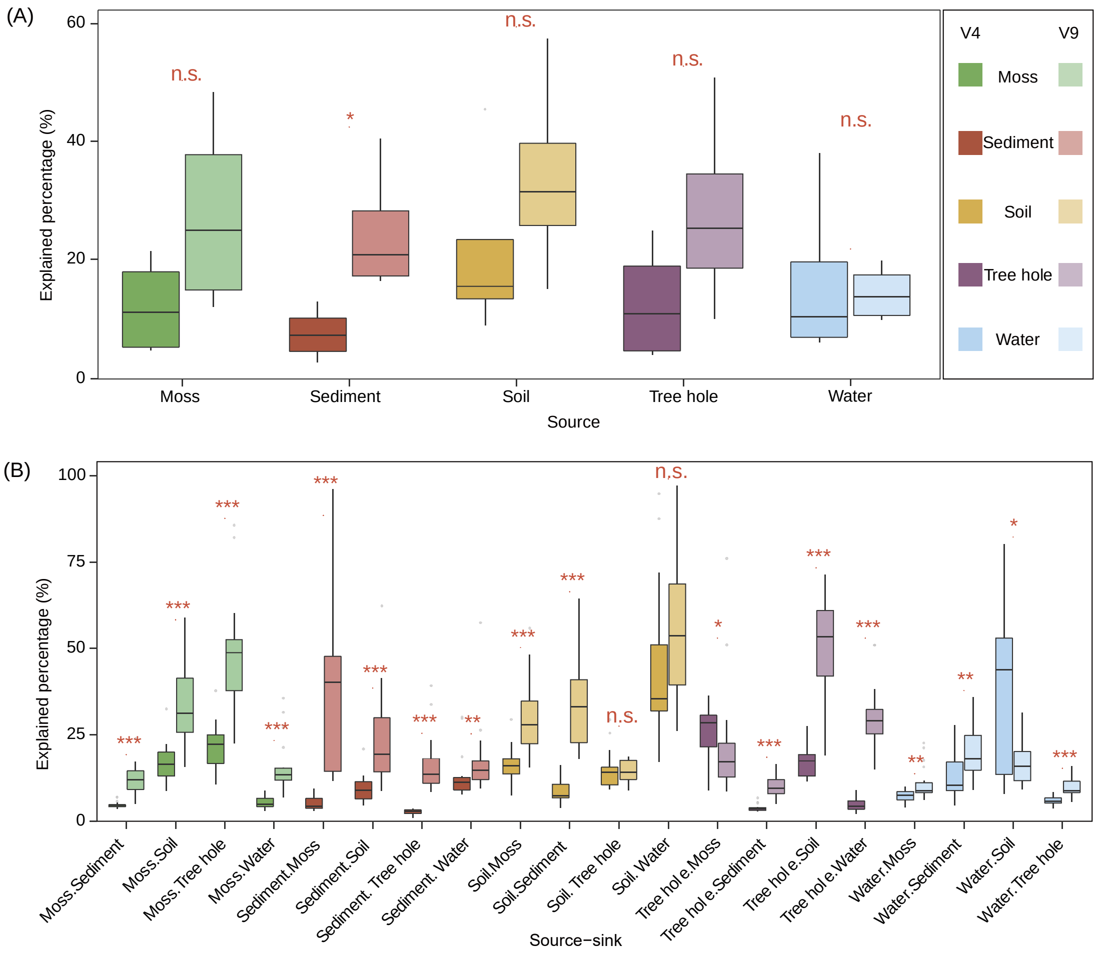

Figure S7 Potential sources of microeukaryotes across different habitats. (**A**) Traceable source (%) for 18S V4 and V9 regions in each habitat, respectively. (**B**) Average traceable source (%) for each habitat to the other four habitats. The statistic is the Wilcoxon test performed in R. The n.s. indicates $p \geq 0.05$. *$p < 0.05$, **$p < 0.01$, ***$p < 0.001$.

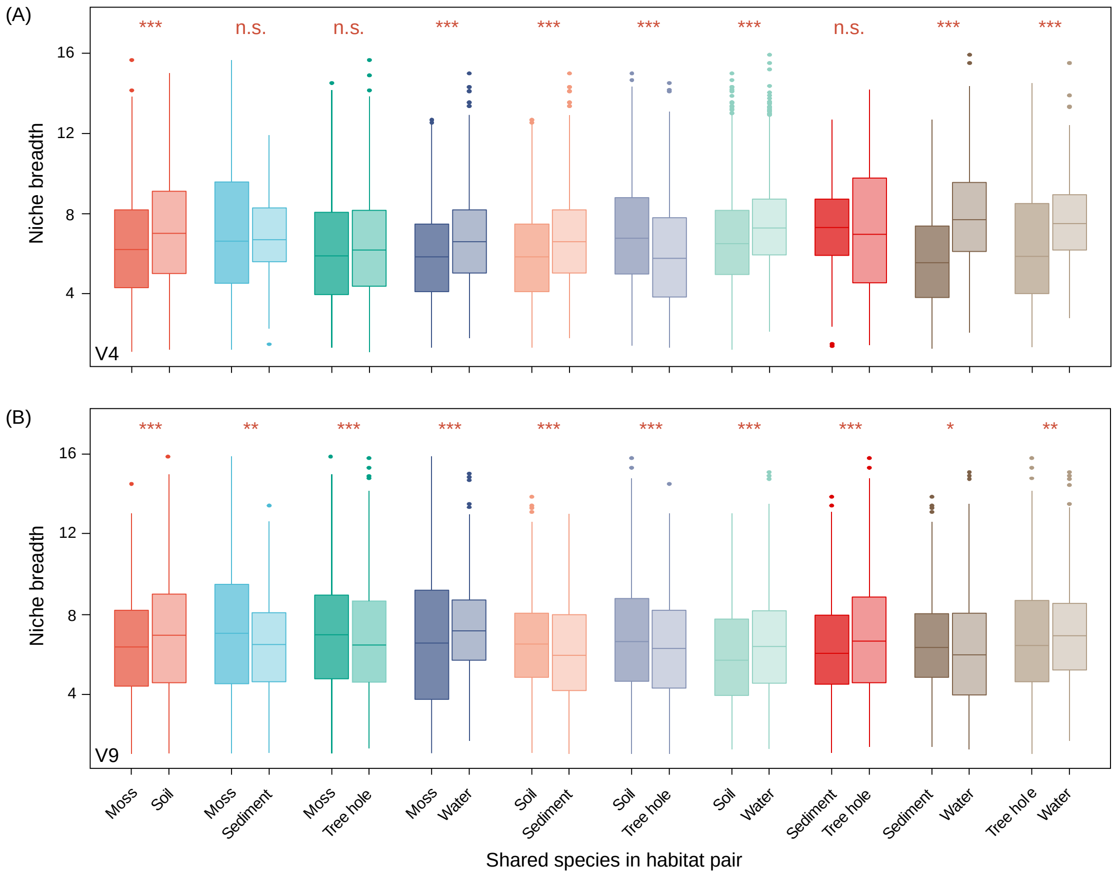

Figure S8 Niche breadth of shared species in each habitat pair based on 18S rRNA gene V4 (A) and V9 (B) regions, respectively. The statistic is based on the Wilcoxon paired test performed in R. The n.s. indicates $p \geq 0.05$. $*p < 0.05$, $**p < 0.01$, $***p < 0.001$.

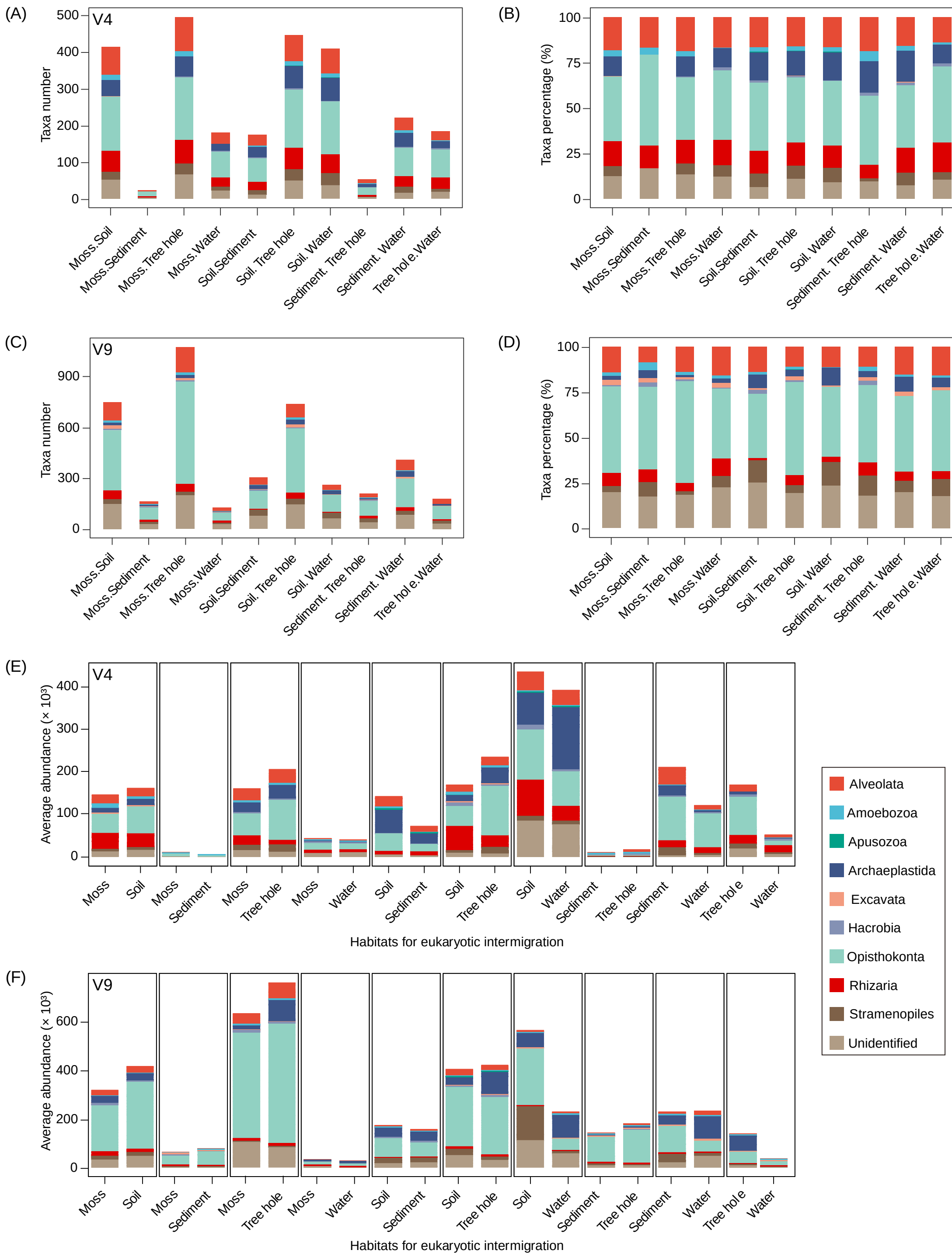

Figure S9 Potential migrated microeukaryotic taxa from 18S rRNA gene V4 (A, B, E) and V9 (C, D, F) regions, respectively. Wilcoxon test was performed to verify whether a zOTU's abundance significantly differs between each pair of habitats. We assume that species may have migrated between the two habitats if there is no significant difference in the abundance of the same species in these two habitats. Numbers (A, C) and relative percentages (B, D) of migrated zOTUs between pairs of habitats. (E, F) Total relative abundance of migrated zOTUs in each pair of habitats. The x-axis is each habitat pair.

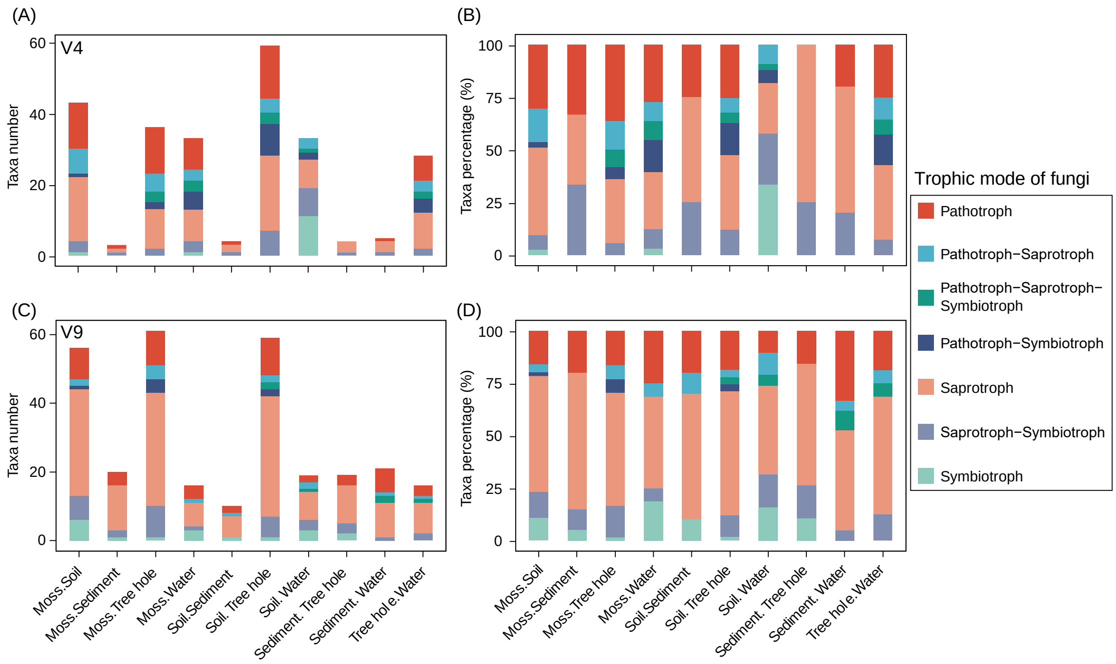

Figure S10 Potential migrated fungal taxa with assigned trophic mode from 18S rRNA gene V4 (A, B) and V9 (C, D) regions, respectively. Numbers (A, C) and relative percentages (B, D) of migrated zOTUs between pairs of habitats.

Table S1 Permutational multivariate analysis of variance (PERMANOVA) using Bray-Curtis dissimilarity based on the supergroup level.

Group	F	R^2
Region (V4 & V9)	106.563	0.222***
Park (6)	4.640	0.048***
Habitat (5)	12.124	0.101***
Region : Park	3.838	0.040***
Region : Habitat	3.819	0.032***
Park : Habitat	3.962	0.165***
Region : Park : Habitat	3.352	0.140***
Residuals	-	0.251
Total	-	1.000

*p < 0.05, **p < 0.01, ***p < 0.001.

Table S2 Observed relative abundances of testate amoeba cells from microscopic analysis, and sequence numbers and relative abundances of testate amoebae from amplifying 18S rRNA gene V4 and V9 regions. Pearson and Spearman correlations of relative abundance are calculated between microscopic analysis and V4-based testate amoeba community.

Genus	Microscope	V4 region		V9 region	
	Relative abundance (%)	Sequence	Relative abundance (%)	Sequence	Relative abundance (%)
Euglypha	16.6	54728	84.582	6818	100
Cryptodifflugia	0.3	218	0.337	0	0
Pseudodifflugia	3.7	426	0.658	0	0
Tracheleuglypha	0.2	28	0.043	0	0
Trinema	9.5	8057	12.452	0	0
Corythion	0.1	58	0.090	0	0
Assulina	2.4	1189	1.838	0	0
Pearson correlation (r)			0.908**		NA
Spearman correlation (r)			0.929**		NA

*$p < 0.05$, **$p < 0.01$, ***$p < 0.001$.

References

1. Stoeck Thorsten, David Bass, Markus Nebel, Richard Christen, Meredith D. Jones, Hans-Werner Breiner, Thomas A. Richards. 2010. "Multiple marker parallel tag environmental DNA sequencing reveals a highly complex eukaryotic community in marine anoxic water." *Molecular Ecology* 19: 21-31. https://doi.org/10.1111/j.1365-294X.2009.04480.x
2. Amaral-Zettler Linda A., Elizabeth. A. McCliment, Hugh W. Ducklow, Susan M. Huse. 2009. "A method for studying protistan diversity using massively parallel sequencing of V9 hypervariable regions of small-subunit ribosomal RNA genes." *PLoS One* 4: e6372. https://doi.org/10.1371/journal.pone.0006372
3. Caporaso J. Gregory, Justin Kuczynski, Jesse Stombaugh, Kyle Bittinger, Frederic D. Bushman, Elizabeth K. Costello, Noah Fierer, et al. 2010. "QIIME allows analysis of high-throughput community sequencing data." *Nature Methods* 7: 335-36. https://doi.org/10.1038/nmeth.f.303
4. Edgar Robert C., Brian J. Haas, Jose C. Clemente, Christopher Quince, Rob Knight. 2011. "UCHIME improves sensitivity and speed of chimera detection." *Bioinformatics* 27: 2194-200. https://doi.org/10.1093/bioinformatics/btr381
5. Team R Core. R: A language and environment for statistical computing. R Foundation for Statistical Computing, Vienna, Austria. https://www.R-project.org/
6. Yu Guangchuang, David K. Smith, Huachen Zhu, Yi Guan, Tsan-Tuk Y. Lam. 2017. "GGTREE: an R package for visualization and annotation of phylogenetic trees with their covariates and other associated data." *Methods in Ecology and Evolution* 8: 28-36. https://doi.org/10.1111/2041-210x.12628
7. Zhang Jinlong. spaa: species association analysis. R package version 0.2.2. https://CRAN.R-project.org/package=spaa
8. Jiao Shuo, Yunfeng Yang, Yiqin Xu, Jie Zhang, Yahai Lu. 2020. "Balance between community assembly processes mediates species coexistence in agricultural soil microbiomes across eastern China." *The ISME Journal* 14: 202-16. https://doi.org/10.1038/s41396-019-0522-9
9. Oksanen Jari, Gavin Simpson, F. Guillaume Blanchet, Roeland Kindt, Pierre Legendre, Peter; hara Minchin, R, Peter Solymos, et al. vegan: community ecology package. https://CRAN.R-project.org/package=vegan
10. Kembel Steven W., Peter D. Cowan, Matthew R. Helmus, William K. Cornwell, Helene Morlon, David D. Ackerly, Simon P. Blomberg, et al. 2010. "Picante: R tools for integrating phylogenies and ecology." *Bioinformatics* 26: 1463-64. https://doi.org/10.1093/bioinformatics/btq166
11. Paradis Emmanuel, Klaus Schliep. 2019. "ape 5.0: an environment for modern phylogenetics and evolutionary analyses in R." *Bioinformatics* 35: 526-28. https://doi.org/10.1093/bioinformatics/bty633
12. Baselga Andrés, C. David L. Orme. 2012. "betapart: an R package for the study of beta diversity." *Methods in Ecology and Evolution* 3: 808-12. https://doi.org/10.1111/j.2041-210X.2012.00224.x
13. Sloan William T., Mary Lunn, Stephen Woodcock, Ian M. Head, Sean Nee, Thomas P. Curtis. 2006. "Quantifying the roles of immigration and chance in shaping prokaryote community structure." *Environmental Microbiology* 8: 732-40. https://doi.org/10.1111/j.1462-2920.2005.00956.x
14. Burns Adam R., W. Zac Stephens, Keaton Stagaman, Sandi Wong, John F. Rawls, Karen Guillemin, Bohannan J. M. Bohannan. 2016. "Contribution of neutral processes to the assembly of gut microbial communities in the zebrafish over host development." *The ISME Journal* 10: 655-64. https://doi.org/10.1038/ismej.2015.142

15. Chen Weidong, Kexin Ren, Alain Isabwe, Huihuang Chen, Min Liu, Jun Yang. 2019. “Stochastic processes shape microeukaryotic community assembly in a subtropical river across wet and dry seasons.” *Microbiome* 7: 138. https://doi.org/10.1186/s40168-019-0749-8

16. Zhou Jizhong, Daliang Ning. 2017. “Stochastic community assembly: does it matter in microbial ecology?” *Microbiology and Molecular Biology Reviews* 81: e00002-e17. https://doi.org/10.1128/MMBR.00002-17

17. Shenhav Liat, Mike Thompson, Tyler A. Joseph, Leah Briscoe, Ori Furman, David Bogumil, Itzhak Mizrahi, et al. 2019. “FEAST: fast expectation-maximization for microbial source tracking.” Nature Methods 16: 627-32. https://doi.org/10.1038/s41592-019-0431-x
